# Gene and Protein Profiles of CHI3L1 and CHI3L2 in Patients with Rheumatoid Arthritis

**DOI:** 10.3390/ph19010101

**Published:** 2026-01-06

**Authors:** Maria Kazakova, Valentina Mihaylova, Zguro Batalov, Rositsa Karalilova, Anastas Batalov, Victoria Sarafian

**Affiliations:** 1 Department of Medical Biology, Medical University-Plovdiv, 4002 Plovdiv, Bulgaria; mariya.kazakova@mu-plovdiv.bg (M.K.);; 2 Research Institute, Medical University-Plovdiv, 4002 Plovdiv, Bulgaria; 3 Department of Propedeutics of Internal Diseases, Medical University-Plovdiv, Blvd. Vasil Aprilov 15A, 4002 Plovdiv, Bulgariaanastas.batalov@mu-plovdiv.bg (A.B.); 4 Clinic of Rheumatology, University Hospital “Kaspela”, 4001 Plovdiv, Bulgaria

**Keywords:** CHI3L1, CHI3L2, rheumatoid arthritis, inflammation, methotrexate, tofacitinib

## Abstract

**Background/Objectives**: Rheumatoid arthritis is an autoimmune disease that induces joint deformity and disability. There are great expectations for biomarkers that would predict the response to treatment. CHI3L1 and CHI3L2 are chitinase-like proteins (CLPs) which lack enzymatic activity. CHI3L1 is expressed by a variety of cells, while reports on CHI3L2 are limited. The aim of the current study is to evaluate gene and protein CHI3L1 and CHI3L2 expressions before and after treatment of patients with RA and to search for correlations with ultrasonography and conventional laboratory parameters. **Methods**: Twenty-four newly diagnosed RA patients (19 females and five males) were enrolled in the study. Fourteen patients were treated with tofacitinib (TOFA) and 10 patients with methotrexate (MTX) for twenty-four weeks. Conventional biochemical and immunological markers were examined at the start of the treatment and after the follow-up period. The activity of RA was assessed via the Disease Activity Score 28 (DAS28). Gene expression and protein analysis were performed. **Results**: Ultrasonographic and clinical laboratory parameters showed improvement after therapy in both groups. A decrease in plasma levels of CHI3L1 (*p* = 0.04 *) and CHI3L2 (*p* = 0.03 *) were found after treatment with TOFA. No changes in either protein level were detected after MTX therapy, nor were any differences discovered in the gene expression of CLPs after treatment with both therapeutics. Strong correlations between CRP, GUS7 and CLPs were also established. **Conclusions**: The similar dynamics of CLPs expression in naïve RA patients and their distinct interplay with disease-related parameters after therapy suggest that both proteins may display different functions in RA pathophysiology.

## 1. Introduction

Rheumatoid arthritis (RA) is a chronic inflammatory multisystemic disease with autoimmune origin that predominantly affects peripheral joints [1]. Despite the progress in understanding the pathophysiology of RA, its etiology remains still unknown. RA provokes joint deformity and disability, as well as reduced life expectancy, due to cardiovascular or pulmonary involvement, infections, or tumors [2]. A pre-RA stage lasting months to years may be characterized by the presence of circulating autoantibodies, increasing concentration of various inflammatory cytokines and chemokines, and altered metabolism. Clinical disease onset involves synovitis and systemic comorbidities affecting the vasculature, metabolism, and bones [3]. There are great expectations for biomarkers that would predict the response to treatment and reduce disease associated cost and unnecessary risk of negative side effects. Furthermore, proteomic biomarkers might support clinicians to diagnose rheumatic diseases earlier and more accurately assess disease activity. These markers should be incorporated into an updated subclassification and further monitored for detection of disease complications [4].

The glycosyl hydrolase family 18 (GH18) proteins are members of a conservative family that exists in different species such as plants, insects, and humans [5]. It contains true chitinases, which degrade chitin polysaccharides, and chitinase-like proteins (CLPs), which bind to but do not degrade chitin. However, their biological roles have recently begun to be clarified. CHI3L1 and CHI3L2 proteins are both CLPs which lack enzymatic activity. Chitinase-3-like 1 (CHI3L1), also known as YKL-40, is expressed by a variety of cells, including macrophages, neutrophils, and epithelial cells [6]. It was shown to participate in different signaling pathways like MAP kinase (MAPK), Akt/protein kinase B, and Wnt/β-catenin signaling [7].

It was detected in situ in inflammatory macrophages associated with rheumatoid synovium [8]. Significantly elevated concentrations of CHI3L1 protein were found in serum and synovial fluid from patients with RA [9] and osteoarthritis (OA) [10] compared to healthy individuals. CHI3L1 levels were significantly higher in synovial fluid from patients with failed total hip arthroplasty than in OA patients [11].

Unlike CHI3L1, which is intensively examined in a variety of pathological conditions, respectively in RA, the reports on CHI3L2 (YKL-39) are limited. Furthermore, no data about the role of CHI3L2 in RA is shown. There is a similarity between CHI3L1 and CHI3L2 in nucleotide sequences, and about 51% homology in amino acid sequences [12]. In fact, only a few papers have examined the expression of CHI3L2 in breast cancer, suggesting a possible role of this protein in carcinogenesis. Previous studies have demonstrated the expression of CHI3L2 in breast cancer and glioblastomas [13]. It is suggested that CHI3L2 is a novel growth and differentiation factor involved in cartilage homeostasis [14]. Parallel data on CHI3L1 and CHI3L2 expression in OA are sparse while in RA they are completely lacking. Even though there are significant improvements in therapeutic approaches for RA, some patients are still non-responders. The possibility of modulating different pathways, molecules, and cells involved in the pathogenesis of RA might help the understanding of lacking or delayed responses to treatment [15]. The inhibitors of the Janus Kinase family (JAK) are introduced as novel drugs for RA patients, such as tofacitinib (TOFA), which is the first JAK-inhibitor approved for RA treatment [16]. Heterogeneous clinical features and different responses to therapy have raised the need to identify predicting molecules or genes reflecting the possible response.

The aim of the current study is to evaluate gene and protein CHI3L1 and CHI3L2 expressions before and after treatment of patients with RA and to search for correlations with ultrasonography and conventional laboratory parameters.

## 2. Results

Twenty-four naïve RA patients who received TOFA or MTX, according to EULAR recommendations, were followed for six months. Summarized information about the clinico-laboratory and ultrasonography parameters are presented in Table 1. The results showed that the levels of all clinico-laboratory markers dramatically increased above the normal laboratory range. DAS28, GUS7, biochemical, and immunological markers improved in both groups 24 weeks after treatment compared with the baseline. The improvement appeared to be less pronounced in the group of patients treated with MTX.

### 2.1. CHI3L1 and CHI3L2 Protein and Gene Levels in Patients on Different Therapy Protocols

**Figure 1:**
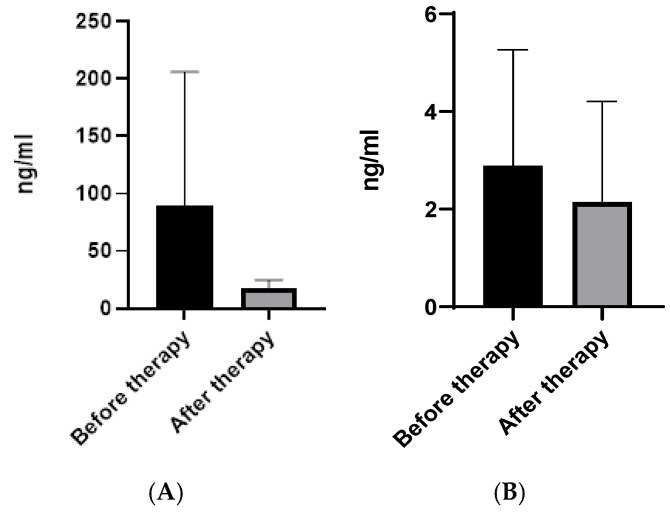
Plasma CHI3L1 (**A**) and CHI3L2 (**B**) levels before and after therapy with JAK inhibitor (TOFA). Legend: Date are presented as mean ± SD.

Plasma and gene expression levels of the two CLPs in RA patients before and after respective therapies were evaluated. A decrease in plasma levels of CHI3L1 (*p* = 0.04 *) (Figure 1A) and CHI3L2 (*p* = 0.03 *) (Figure 1B) was found after treatment with JAK-inhibitors.

**Figure 2:**
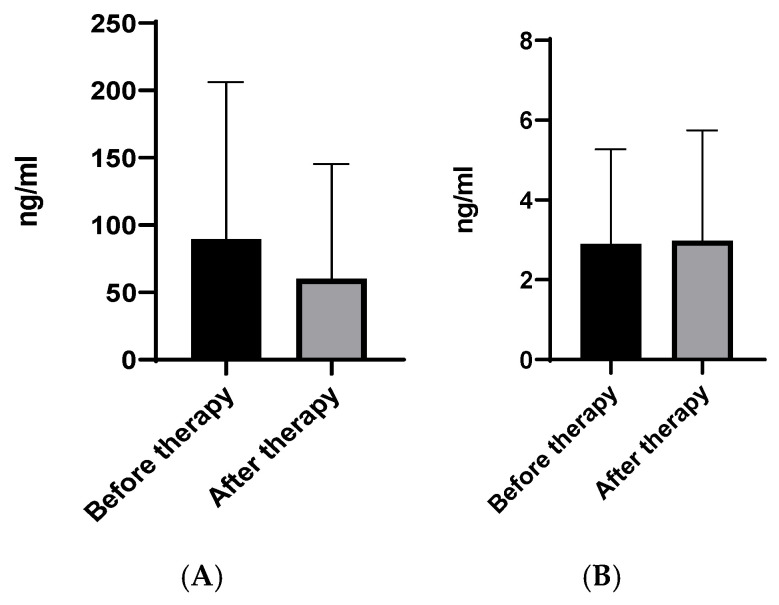
Plasma CHI3L1 (**A**) and CHI3L2 (**B**) levels before and after therapy with MTX. Legend: Date are presented as mean ± SD.

No changes in either protein level were detected after MTX therapy (Figure 2). Despite there being no significant variations in CPL concentrations after receiving MTX, the levels of CHI3L1 decreased more rapidly compared to CHI3L2 (*p* = 0.06).

Furthermore, no differences in the gene expression of CHI3L1 and CHI3L2 after the treatments with both therapeutics were found. 

### 2.2. Correlation Between Chitinase-like Proteins, Ultrasonography Scores and Biochemical Markers

**Figure 3:**
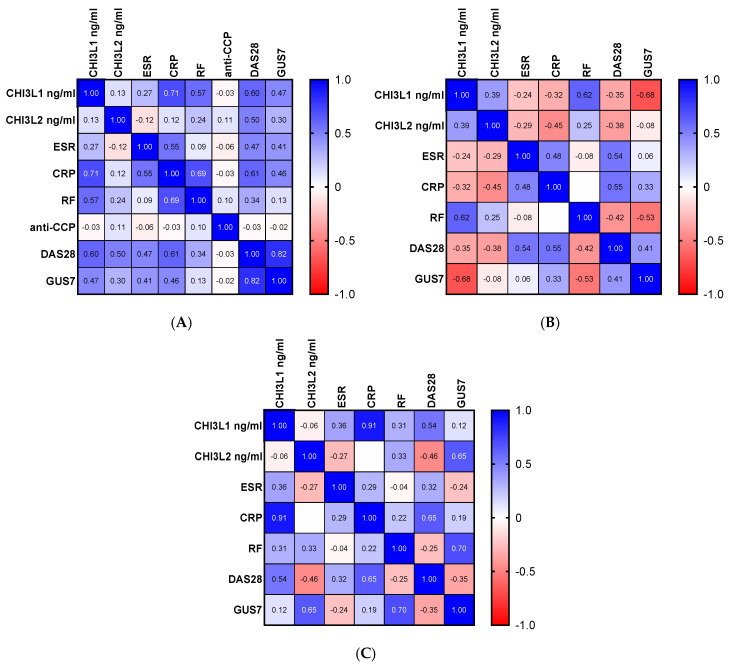
Correlation between CLPs, ultrasonography scores and biochemical markers before (**A**) and after therapy with TOFA (**B**) and MTX (**C**). Legend: The numerical values reflect the correlation r coefficient. The sign (-) should be read as minus.

Possible associations before and after treatment between the main variables of interest were analyzed. A strong positive correlation was found between CHI3L1 and DAS28 (*p* = 0.002, z = 0.600), CHI3L1 and CRP (*p* = 0.0004, z = 0.710), and CHI3L1 and RF (*p* = 0.003, z = 0.570) before therapy (Figure 3A). A significant relationship between CHI3L1 and RF (*p* = 0.019, z = 0.620) and CHI3L1 and GUS7 (*p* = 0.007, z = −0.680) after therapy with JAK-inhibitors was detected (Figure 3B). Correlations between CHI3L1 and CRP (*p* = 0.0004, z = 0.910), as well as between CHI3L2 and GUS7 (*p* = 0.041, z = 0.650), were discovered in the RA patient group after therapy with MTX (Figure 3C).

Just like CHI3L1, CHI3L2 showed an association with DAS28 (*p* = 0.002, z = 0.597) in RA patients before therapy. No relationship between the investigated parameters and CHI3L2 after therapy was observed.

## 3. Discussion

The expression of CHI3L1 and CHI3L2, two members of the chitinase-3-like family, has not been examined in parallel in naïve RA patients so far. Studies by our team and others have demonstrated that YKL-40/CHI3L1 contributed to ongoing inflammation, tissue remodeling and tumor development [17]. CHI3L1 was related to tissue remodeling [8], monocyte to macrophage maturation during inflammation, and tumor processes [18]. As CHI3L1 levels correlated with the severity of synovial inflammation but not with OA grading, it is assumed that its levels in synovial fluid and serum reflect the inflammatory state of the joint rather than cartilage degradation. Several studies suggested that CHI3L1 might be a candidate marker for the diagnosis, prognosis, and monitoring of osteoarthritic and rheumatoid disease [11,19]. In this context, our results are informative and support the involvement of CHI3L1 in the pathogenesis of RA. We determined higher CHI3L1 levels before therapy and significant improvement of all clinico-laboratory markers after therapy. Furthermore, a strong correlation with biochemical, immunological and ultrasonography parameters was revealed. In addition, in our previous study, plasma CHI3L1 concentrations in RA patients were detected to be significantly increased compared to the levels in healthy individuals [9]. Since the results for CHI3L1 expression were definite and predictable, the protein and gene expression of CHI3L2 were, interestingly, also elevated, similarly to the CHI3L1 pattern.

It was reported that CHI3L2 is secreted by chondrocytes in vitro [20]. In contrast to CHI3L1, which is widely recognized as a general marker of inflammation and tissue remodeling, CHI3L2 has been less extensively studied but appears to be more closely associated with macrophage differentiation and localized inflammatory responses. While CHI3L1 demonstrates robust diagnostic and prognostic performance across a broad range of inflammatory conditions, CHI3L2 may offer added clinical value by reflecting disease-specific immune activation patterns. Therefore, CHI3L2 should be considered a complementary biomarker rather than a replacement for CHI3L1, potentially improving risk stratification or disease characterization when used in combination. Recently, it was shown that patients with OA or RA develop autoimmune reactions against CHI3L2 [21]. No data has yet reported whether this molecule could also be a marker for arthritic joint disease. On the other hand, it was proved that CHI3L2 is expressed both in the cytoplasm of cancer cells and macrophages. The authors suggested that the protein might regulate STAT-3 and ERK1/2 phosphorylation in breast cancer cell lines [22]. There is no available scientific data on the involvement of CHI3L2 in inflammatory diseases. In this respect, our study provides new results for the association of CHI3L2 with joint inflammation. Although traditional biomarkers such as CRP, DAS28, and RF remain essential for disease assessment, CHI3L2 may offer complementary clinical information by reflecting macrophage-specific inflammatory activity. This additional perspective could be useful for identifying patient subgroups with distinct inflammatory profiles or for monitoring response to targeted therapies, such as JAK inhibitors. While these potential applications are preliminary, they highlight the added value of considering CHI3L2 alongside established markers.

Ultrasonography and the GUS7 illustrate soft tissues and/or bone erosions and are implemented in routine clinical practice for monitoring disease activity [23]. The advantages of US assessment are early detection and excellent monitoring of subtle progression of arthritic alterations [24]. In our study, a correlation between chitinase-like-proteins and the results from sonography was determined. However, CHI3L1 and CHI3L2 may not be specific for articular chondrocytes, but they may serve as potential markers for the assessment of arthritic joints. The research group of Steck et al. found that CHI3L2, in comparison with CHI3L1, was up-regulated in response to cartilage degeneration in late OA. They suggested that the higher CHI3L1 protein levels in synovial fluid and serum from patients with joint disease were derived from cells of the synovial membrane rather than from cartilage. Therefore, they do not reflect cartilage metabolism in late OA stages [25]. JAK inhibitors are intracytoplasmic protein tyrosine kinases that bind to the cytoplasmic region of transmembrane cytokine receptors. They act as a signal transducer and activator of transcription (STAT) [26]. It was defined that JAK/STAT signaling could block multiple cytokines related to RA [27]. As CHI3L1 was determined to be activated by pro-inflammatory cytokines such as IL-1, TNF-α and IL-6, we could speculate that the therapy with JAK inhibitors directly decreases the levels of CHI3L1. On the other hand, the efficacy of MTX in RA is due to multiple mechanisms of action [28]. It was found that MTX could significantly reduce the level of pro-inflammatory cytokines through inhibition of immune cells in RA synovium [29]. Obviously, MTX decreased the rate of inflammation indirectly by acting first on the cells that secrete cytokines, but not through direct cytokine–cytokine receptor interactions. We assume that these facts could explain the slower response to the therapy with MTX in comparison with the action of JAK inhibitors.

The differential response observed between TOFA and MTX raises the question of whether CLPs such as CHI3L1 and CHI3L2 could serve as biomarkers for treatment stratification. Given the central role of JAK–STAT signaling in inflammatory pathways and macrophage activation, modulation of CHI3L1 and CHI3L2 levels may reflect sensitivity to JAK inhibitor therapy. Although the present study was not designed to assess predictive utility, these findings suggest that CLPs could potentially complement existing markers in identifying patients more likely to respond to JAK inhibition, a hypothesis that warrants validation in larger, prospective studies.

Our results showed significantly higher plasma CHI3L1 and CHI3L2 levels in naïve RA patients, but no important changes at the mRNA level. We could propose that CLP expressions might be regulated post-transcriptionally. In previous studies, a new regulatory axis lncRNAs/miR-30e/CHI3L1 was proven in systemic sclerosis [13]. It is quite possible that CHI3L2, as well as CHI3L1, is also post-transcriptionally regulated in this pathology. One plausible pathway is microRNA-mediated regulation, which is known to modulate mRNA stability and translational efficiency in immune cells. In addition, post-translational processes such as protein folding, secretion, and extracellular stability may further influence measurable CLP concentrations. Although these mechanisms were not directly examined in the present study, they provide a biologically reasonable explanation of our findings.

Although innovative in terms of design, molecules, and target groups examined, our study has its limitations. The relatively small sample size (*n* = 24) represents an important limitation of this study, as it restricts statistical power and limits the generalizability of the findings. Consequently, the results should be interpreted with caution and considered exploratory, requiring confirmation in larger, adequately powered cohorts. Next, the follow-up period could be extended. In addition, this study may be influenced by potential confounding factors such as baseline disease severity, disease duration, and inter-individual variability in inflammatory burden. These factors could have affected both biomarker levels and treatment responses and should be considered when interpreting the results. Also, we examined CHI3L1 and CHI3L2 in plasma only. The lack of synovial fluid measurements represents an important limitation, as it prevents direct assessment of joint-specific inflammatory processes and may limit the interpretation of circulating biomarker levels in relation to local pathology.

In conclusion, we evaluated the protein and gene expression levels of CHI3L1 and CHI3L2 in RA patients before and 24 weeks after therapy with TOFA and MTX. Both CLPs showed a similar pattern of expression, with CHI3L1 being present in higher levels both as a transcript and a protein. The improvement of all disease-related markers was less significant in the group treated with MTX therapy than in the patients with JAK inhibitors.

## 4. Materials and Methods

### 4.1. Patients

Twenty-four newly diagnosed RA patients according to the ACR/EULAR 2010 criteria [30] at the Rheumatology Clinic of University Hospital “Kaspela’’ were enrolled in the study. The mean age of the participants (19 females and five males) was 55 ± 2 years. Fourteen patients who met the EULAR treatment recommendations for the management of RA with synthetic and biological disease-modifying antirheumatic drugs [31] received the JAK inhibitor (TOFA), and 10 patients received MTX. The follow up period was 24 weeks after the application of therapy. Conventional biochemical and immunological markers such as rheumatoid factor (RF) and anti-CCP-antibodies, as well as acute phase reactants-erythrocyte sedimentation rate (ESR) and C- reactive protein (CRP), were examined at the start of the treatment and after the follow-up period. Venous blood samples were obtained before and after therapy (24 weeks). TOFA and MTX were administered in their optimally approved daily doses. The study was approved by the University Ethics Committee (Protocol№ 3/31 May 2018). Informed consent was signed by all examined individuals according to the Helsinki Declaration.

All patients were examined to assess the activity of RA via the Disease Activity Score 28 (DAS28), which evaluates 28 joints. It is based on counts of tender and swollen joints, the patient’s global assessment of disease activity, and ESR or CRP. The criteria for separating remission, low, moderate, and high disease activity are scores of 2.6, 3.2, and 5.1, respectively [32].

### 4.2. Methods

Isolation of plasma and white blood cells (WBCs), total mRNA extraction, and cDNA synthesis were performed following the protocols used in our previous publications [33].

### 4.3. Ultrasound Examination

Ultrasound assessment of wrist, hands, and forefoot was conducted using a GE Logic E9 machine (GE HealthCare, Chicago, IL, USA) with a ML6-15-D Matrix Array linear probe for the joint/tendon assessment. Two-dimensional US (B-Mode/Gray scale US) and Power Doppler US were performed [34]. GSUS frequency was 11–15 MHz depending on the examined joint and GSUS gain was estimated based on joint regions and patients with an average value of 50%. The following settings were used for PDUS: frequency 8.3 MHz; pulse repetition frequency 600–800 Hz; PDUS gain in relation to joint regions and patients with an average value of about 50%; and low wall filter. The German US7 score of Backhaus et al. [35] was applied.

Using GSUS and PDUS, we examined seven joints of the clinically dominant hand/foot, affected more by swelling or tenderness, based on the German US7 score (wrist, second and third MCP and PIP, and second and fifth metatarsophalangeal (MTP) joints), according to the approach already published [36].

### 4.4. qPCR Assessment of Gene CHI3L1 and CHI3L2 Expression

For the quantitative measurement of CHI3L1 and CHI3L2, mRNA gene expression analysis was conducted. The primer combinations used for the longest RNA transcripts of CHI3L1 and CHI3L2 were synthesized by Integrated DNA Technologies (Leuven, Belgium). The sequences were as follows: for CHI3L1 (Fw5′CTGCTCCAGTGCTGCTCT-3′, Rev5′-TACAGAGGAAGCGGTCAAGG-3′), and for CHI3L2 (Fw5′GCCGCGACGCGTGACCAGAAGTCTCTCTGGGC; Rev5GCCGCGGCGGCCGCCCAAGGAGC CAAGGCTTCTC3′). RNA normalization was performed using several internal controls: GAPDH (Fw 5′-AGG TCCACCACTGACACGTTG-3′, Rw5′-AGCT-GAACGGGATGCTCACT-3′), ACTIN (Fw 5′-AGTGTGACG TGGA-CATCCGGA-3′, Rev 5′-GCC AGGGCAGTGATCTCCTCCT-3′), and hUBC (Fw 5′-TCCTCAGGCAGAG-GTT GATCTT-3′, Rev 5′-GGACCAAGTGCA-GAGGTGGACTCTT-3′) (Integrated DNA Technologies, Leuven, Belgium). The qPCR reactions were run on Rotor-Gene Q 600 (Qiagen, Hilden, Germany). Quantitative evaluations were performed by the 2−DDCt method. Each sample was analyzed in duplicate.

### 4.5. ELISA for Detection of Plasma Levels of CHI3L1 and CHI3L2

Plasma levels of CHI3L1 (Elabscience Human GP39 YKL-40, Cat NoE-EL-H0037 Lot No CV0, Houston, TX, USA) and CHI3L2 (Circulex Human YKL-39 CAT No CY-8087, MBL, Woods Hole, MA, USA) were measured for all patients by ELISA. The tests were performed in duplicate following the manufacturer’s instructions. The intra-assay coefficients of variation (CV) were 10%, and the inter-assay CV were <12%. The assay employs the sandwich-based ELISA method with optical density measured at 450 nm on Tecan sunrise ELISA reader.

### 4.6. Statistical Analysis

The data obtained were processed statistically using the Prism software, version 10. Descriptive statistics were applied to determine group means and data parametricity. A *t*-test of related variables was then employed to determine statistical significance in both groups. Because of the exploratory nature of the study and the relatively small sample size, no formal correction for multiple testing (e.g., Bonferroni or false discovery rate) was applied. Pearson’s correlation coefficient was calculated to evolute correlation between studied variables. Statistical significance levels of the null hypothesis were *p* < 0.05.

## 5. Conclusions

The similar dynamics of CHI3L1 and CHI3L2 expression in naïve RA patients and their distinct interplay with disease-related parameters after therapy suggest that both proteins may display different functions in RA pathophysiology.

## Figures and Tables

**Table 1:** Clinico-laboratory parameters in RA patients before and after therapy.

Parameters	Patients Before Therapy (*n* = 24)	Patients After Therapy with TOFA (*n* = 14)	Patients After Therapy with MTX (*n* = 10)
CRP mg/L, (normal range ≤ 6)	20.45 ± 5.44	9.9 ± 3.6	10.17 ± 3.35
RF IU/mL, (normal range < 10)	172 ± 44.29	70 ± 11.5	87.18 ± 29
ESR mm/h, (normal range 15–20)	53.68 ± 4.11	27 ± 9	32.7 ± 4.9
DAS28, remission (≤2.6)	5.66 ± 0.2	3.6 ± 0.2	3.9 ± 0.2
GUS7, (0–108)	26.5 ± 2.4	11 ± 1	14 ± 1.8

Legend: Data are presented as the median ±  standard deviation by Graph Pad Prism-10. CRP—C-reactive protein, RF—rheumatoid factor, ESR—erythrocyte sedimentation rate, DAS28—Disease activity score 28, GUS7—Ultrasound score.

## Data Availability

Data is contained within the article.
